# Health-care associated infections. Patient characteristics and influence on the clinical outcome of patients admitted to icu. envin-helics registry data

**DOI:** 10.1186/2197-425X-3-S1-A82

**Published:** 2015-10-01

**Authors:** X Nuvials, M Palomar, F Alvarez-Lerma, P Olaechea, S Otero, S Uriona, M Catalán, R Gimeno, MP Gracia, I Seijas, ENVIN-HELICS

**Affiliations:** Hospital Universitari Arnau de Vilanova, IRB Lleida, Lleida, Spain; Hospital Universitari Arnau de Vilanova. IRB Lleida, Intensive Care Medicine, Lleida, Spain; Hospital del Mar, Intensive Care Medicine, Barcelona, Spain; Hospital de Galdakao, Intensive Care Medicine, Galdakao, Spain; Hospital Universitari Vall d'Hebron, Medicina Preventiva, Barcelona, Spain; Hospital 12 de Octubre, Intensive Care Medicine, Mardrid, Spain; Hospital Univesitario La Fe, Intensive Care Medicine, Valencia, Spain; Hospital de Cruces, Intensive Care Medicine, Baracaldo, Spain

## Introduction

Health care associated infections (HCAI) are frequent in patients admitted to ICU and have a great impact on the clinical outcome.

## Objectives

To analyze the characteristics of patients developing HCAI during ICU admission and their influence on the clinical outcome.

## Methods

Prospective, observational, multicenter and voluntary enrollment study (Spanish registry ENVIN-HELICS) [1]. All patients admitted to ICU for > 24 hours between 1st April and 30th June during the period from 2006 to 2013 were included. All episodes of HCAI were recorded during the follow-up. HCAI were categorized as Ventilator Associated Neumonia (VAP), Primary bacteriemia and Catheter related bacteriemia (PB-RCB) and catheter-associated Urinary Tract Infection (CAUTI) according the HELICS definitions [2]. Patients were categorized as: no infection (NI) when they had not infection during admission, no-HCAI when they had infection not related with health-care during admission, and HCAI when they had at least 1 episode of HCAI. Demographic data, risk factors, length of stay and mortality were recorded. Univariate analysis was done using Chi-square test. p value < 0,05 was considered statistical significant.

## Results

Among 129,037 patients admitted to ICU, 58,706 infections were recorded, of whom 15,490 (26.4%) were HCAI: 6,068 (10.3%) VAP, 4,943 (8.4%) CAUTI and 4,479 (7.6%) PB-CRB. 12,612 (82.1%) of HCAI were ICU acquired, 1,966 (12,7%) were hospital acquired and 686 (4,4%) were acquired in the community. Table 1 shows demographic data, risk factors and the clinical outcome of patients accordingly the infectious status.

**Table 1:** Table 1

	No infection (n = 89.838)	non HCAI (n= 27.850)	HCAI (n= 11.349)	p
Age: average ± SD	62.5 ± 16.4	62.6 ± 16.3	60.9 ± 16.3	P < 0.05
Sex: n(%)male Female	58,979 (65.6) 30,850 (34.4)	18,115 (65.0) 9,735 (35.0)	7,292 8 (65.2) 3,956 (34.8)	NS
APACHE II: average ± SD	12.53 ± 7.5	18.6 ± 8.2	19.6 ± 8.0	P < 0.05
Type of admission: n(%) Coronary Medical Elective surgery Emergency surgery Trauma	27,363 (92.5) 29,506 (55.4) 20,268 (87.6) 5,467 (43.5) 6,146 (67.6)	1,417 (4.8) 17,204 (32.3) 1,859 (8.0) 5,705 (45.5) 1,395 (15.4)	793 (2.7) 6,514 (12,2) 1,004 (4.3) 1,387 (11.0) 1,546 (17.0)	P < 0.05
Antimicrobial treatment at admission (n= 28.343): n (%)	10,878 (38.4)	13,228 (46.7)	4,237 (14.9)	P < 0.05
Extrarenal depuration technic (n = 6.400): n(%)	2,163 (33.8)	2,509 (39.2)	1,728 (27.0)	P < 0.05
Parenteral Nutrition (n = 14.565): n(%)	5,064 (34.8)	6,069 (41.7)	3,432 (23.6)	P < 0.05
Mortality: n (%)	5,764 (6,4)	4,966 (17.8)	2,976 (26.2)	P < 0.05
Length of stay: days ± SD	4.9 ± 4.8	10.1 ± 9.4	22.8 ± 16.2	P < 0.05

## Conclusions

Patients admitted to ICU with HCAI have a worse clinical outcome( higher mortality and length of stay), and are more severely ill on admission than patients with non related HCAI.
